# Molecular mechanisms of hypoxia in cancer

**DOI:** 10.1007/s40336-017-0231-1

**Published:** 2017-05-11

**Authors:** Amarnath Challapalli, Laurence Carroll, Eric O. Aboagye

**Affiliations:** 1 Department of Clinical Oncology, Bristol Cancer Institute, Horfield Road, Bristol, United Kingdom; 2 Department of Surgery and Cancer, Imperial College, GN1, Commonwealth Building, Hammersmith Hospital, Du Cane Road, London, W120NN United Kingdom

**Keywords:** Hypoxia, MRI, Positron emission tomography, Hypoxia radiotracers

## Abstract

**Purpose:**

Hypoxia is a condition of insufficient oxygen to support metabolism which occurs when the vascular supply is interrupted, or when a tumour outgrows its vascular supply. It is a negative prognostic factor due to its association with an aggressive tumour phenotype and therapeutic resistance. This review provides an overview of hypoxia imaging with Positron emission tomography (PET), with an emphasis on the biological relevance, mechanism of action, highlighting advantages, and limitations of the currently available hypoxia radiotracers.

**Methods:**

A comprehensive PubMed literature search was performed, identifying articles relating to biological significance and measurement of hypoxia, MRI methods, and PET imaging of hypoxia in preclinical and clinical settings, up to December 2016.

**Results:**

A variety of approaches have been explored over the years for detecting and monitoring changes in tumour hypoxia, including regional measurements with oxygen electrodes placed under CT guidance, MRI methods that measure either oxygenation or lactate production consequent to hypoxia, different nuclear medicine approaches that utilise imaging agents the accumulation of which is inversely related to oxygen tension, and optical methods. The advantages and disadvantages of these approaches are reviewed, along with individual strategies for validating different imaging methods. PET is the preferred method for imaging tumour hypoxia due to its high specificity and sensitivity to probe physiological processes in vivo, as well as the ability to provide information about intracellular oxygenation levels.

**Conclusion:**

Even though hypoxia could have significant prognostic and predictive value in the clinic, the best method for hypoxia assessment has in our opinion not been realised.

## What is hypoxia?

Hypoxia generally refers to sub-physiologic tissue oxygen levels (<5–10 mmHg). Tumour hypoxia, a hallmark of malignancy, is a common and important feature of the tumour microenvironment. It is the consequence of an oxygen delivery versus consumption mismatch that occurs when cell proliferation outstrips neoangiogenesis during tumour growth. This results in very low oxygen levels (<5 mmHg) in tumours versus 40–60 mmHg in healthy tissues [1]. Hypoxia can generally be classified as (1) perfusion-related (acute) hypoxia due to insufficient blood flow, (2) diffusion-related (chronic) hypoxia caused by an increase in diffusion distances with tumour expansion, and (3) anaemic hypoxia caused by a decrease in oxygen transport capacity [2]. The latter two are considered relatively stable, whereas the degree of acute hypoxia may change in a short time. Cancer cells respond differently to decreased oxygen tension by eliciting cell death or cell survival, which partially depends on the time of exposure to hypoxia.

The origin of chronic hypoxia in human tumours was postulated by Thomlinson and Gray in 1955 [3]. Chronic hypoxia, also referred to as diffusion-limited hypoxia (DLH), is caused by consumption of oxygen by cells close to vessels, leaving inadequate oxygen for the cells further away from the vessels (>100 µm of capillary blood vessels), as demonstrated by means of phosphorescence lifetime imaging of R3230AC tumours in dorsal flap window chambers [4]. Chronic hypoxic changes are exacerbated in larger tumours and contribute to long-term cellular changes such as high frequency of DNA breaks, accumulation of DNA replication errors, potentially leading to genetic instability and mutagenesis [5, 6].

Brown and colleagues [7] were the first to present a second form of hypoxia: acute hypoxia. Acute hypoxia is an abrupt and brief exposure to short-term hypoxia (between a few minutes and up to 72 h) which occurs consequent to fluctuations in tumour perfusion accompanying functionally and structurally defective vascular network in tumour (overdilated, hyperpermeable, tortuous, and disrupted), and associated with high-interstitial pressure of the extracellular matrix [8]. This leads to periods of better or worse oxygenation [9, 10] promulgating the lexicon—cycling hypoxia [11]. Temporal occlusion of blood vessels caused by blood clots or tumour emboli can also cause acute hypoxia [12]. Acute hypoxia can lead to generation of high levels of reactive oxygen species (ROS) that can damage cells [13]. Cellular adaptations to these conditions have been enumerated and include decreasing oxidative metabolism and activating autophagy [14, 15]. Increased radio-resistance of cancer cells [13, 16], induction of spontaneous metastasis [10, 17, 18], and genomic instability due to delayed DNA damage response and rapid p53-dependent apoptosis [19, 20] can also result from hypoxia, leading to an aggressive tumour phenotype.

Hypoxia represents a unique tumour vulnerability to be exploited in the context of newly emerging personalised medicine strategies. Undoubtedly, both chronic and acute tumour hypoxia directly affect clinical responses to therapy by influencing tumour growth, ability to metastasize, and resistance to cell death.

## Methods

A comprehensive PubMed literature search was performed, identifying articles relating to types of hypoxia, biological significance of hypoxia, measurement of hypoxia, MRI methods, and PET imaging of hypoxia in preclinical and clinical settings, up to December 2016. Search terms that were used to identify such articles were “hypoxia imaging,” “MRI,” “FMISO”, “FAZA”, “FETNIM”, “EF5”, “HX4”, “RP-170”, “Cu-ATSM”, and “PET” or “positron emission tomography.” Original publications in English were selected for inclusion in this review.

### Biology and clinical significance of hypoxia

Tumour hypoxia is frequently seen in solid tumours, and tumour cells survive by activating different signalling pathways leading to a plethora of temporally or spatially heterogeneous changes in tumours (Table 1), elicited at different thresholds of oxygen tension [21–52]. In fact, during malignant growth, hypoxic regions are associated with increased genetic instability and more aggressive phenotype which correlate with tumour metastasis risk. Likewise, hypoxia causes unequivocal resistance to cancer treatments, such as reduced drug penetration, intrinsic chemoresistance (by mechanisms including loss of sensitivity to p53-mediated apoptosis or diminution of cell proliferation by metabolic stress), and resistance to ionizing radiation (reduced ability of oxygen to fix DNA lesions).

**Table 1:** Signalling pathways activated by tumour hypoxia promoting cell survival

Signalling pathways	Comments
Hypoxia inducible factor (HIF1) [21–27, 38–51]	Mediates tumour cell responses to hypoxia
	Glucose metabolism HIF1alpha regulates the switch from pyruvate catabolism and oxidative phosphorylation to glycolysis in both hypoxic and normoxic cells, by activating the expression of glucose transporters (GLUT 1 and 3) and glycolytic enzymes [39, 44, 47]
	Lipid metabolism HIF2 regulates fatty acid metabolism and induces significant changes in the expression of glycolipids and glycoproteins [51].
	DNA repair Tumour hypoxia increases mutation rate and decreases DNA repair resulting in genetic instability Acute hypoxia can result in high levels of reactive oxygen species (ROS), which causes DNA damage and malignant progression upon reoxygenation [38, 43] Chronic hypoxia can also lead to accumulation of DNA replication errors or DNA breaks over time [49]
	Apoptosis HIF1 initiates hypoxia-mediated apoptosis (during prolonged severe hypoxia) by enhancing the expression of the several genes such as Bcl-2, p53, BNIP3, and BNIP3L [48].
	Angiogenesis Hypoxia induces the imbalance between pro- and anti-angiogenic factors’ production, which results in chaotic blood vessel formation. HIF1 is involved in all steps of blood vessel formation [40] by contributing to i) Endothelial progenitor cell (EPC) recruitment and differentiation into endothelial cells (ECs), by VEGF, FGF & PDGF regulation, [42] ii) induction of matrix metalloproteinases (MMPs) and iii) recruiting smooth muscle cells and pericytes to stabilise blood vessels [45]. However, in tumours, new blood vessels are often abnormal, immature, leaky, and dysfunctional, resulting in hypoxia [40] [41]
	Metastases Contributes to metastases by altering cancer cell adhesion and motility [41], through regulation of epithelial-to-mesenchymal transition (EMT), which is characterised by a decrease in epithelial-associated and an increase in mesenchymal-associated gene expression [50], promotes migration and invasion abilities through induction of CXCR4, CA9, MMP [46]
Unfolded protein response (UPR) [28–32]	This is an oxygen-sensitive signalling pathway mediating cell survival under hypoxic conditions UPR restores homeostasis by alleviating the ER stress due to accumulation of misfold proteins under hypoxic conditions This is mediated through protein kinase R–like endoplasmic reticulum kinase (PERK), inositol-requiring protein 1 (IRP-1), and activating transcription factor 6 (ATF6), which induce hypoxia-associated metastases and radioresistance
AKT-mTOR pathway [28]	Mediates cell survival under hypoxia Hypoxia-induced inhibition of mTOR-complex will induce autophagy, similar to the ER-stress-induced UPR
Other down stream changes	
miRNAs [32]	These interact with target mRNA’s thereby suppressing target gene and consequent protein expression, thus regulatingproliferation, apoptosis, angiogenesis and DNA repair
Epigenetic changes [33, 34]	Chromatin alterations such as histone acetylation/deacetylation allow cells to adapt to hypoxic stress
p53 [35]	TH is one of the earliest driving forces which leads to loss of p53 function during tumourigenesis leading to treatment resistance
Metabolic changes [36, 37]	Hypoxia causes tumour cells to switch to glycolysis for energy production (due to decrease in mitochondrial oxidation) Glycolytic products such as pyruvate and lactate induce HIF1alpha accumulation (Feed forward mechanism)

A number of biological consequences of low oxygen levels have been elegantly described by Höckel and Vaupel [53]. At pO_2_ levels less than 10–15 mmHg, cells become radioresistant and gene expression of hypoxia-regulated genes under control of hypoxia-inducible factor (HIF1) increases. Decreased adenosine triphosphate (ATP) synthesis is seen at pO_2_ levels less than 10 mmHg and together with decreased protein synthesis leads to lower oxygen consumption by cells. Finally, pO_2_ levels less than 1 mmHg reduce oxidative phosphorylation and conversely enhance glycolysis to maintain adequate ATP levels [54].

#### Role of HIF1alpha

**Fig. 1:**
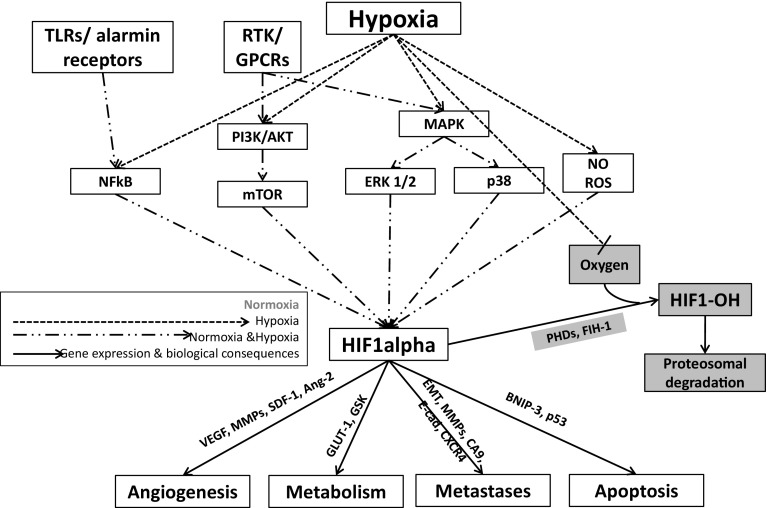
Regulation of HIF1alpha in normoxic and hypoxic conditions and biological consequences of hypoxia

Pathological hypoxia is a common microenvironment factor in tumours that facilitates cell survival and propagation of the tumour. The cross-talk between tumour and its microenvironment is essential for tumour survival [55]. Hypoxia-inducible changes not only affect tumour cells but also the tumour microenvironment [56]. Hypoxia-inducible factor 1 and 2 (HIF1 and HIF2, respectively) are oxygen-sensitive, heterodimeric transcription factors that act as key mediators of the cellular adaptation to low oxygen. HIF1 regulates glycolysis and pyruvate metabolism, and HIF-2 controls fatty acid metabolism. HIF1 is a heterodimeric protein consisting of HIF1alpha (oxygen regulated) and HIF1beta (constitutively expressed) dimers. Hypoxia stabilises HIF1alpha which stimulates expression of a variety of genes controlling metabolic pathways, pH regulation, angiogenesis, metastatic potential, DNA replication, protein synthesis, and treatment resistance, which (1) enhances cell survival via growth factor signalling and inhibition of pro-apoptotic pathways, (2) contribute to tumour neovascularization via VEGF, VEGF receptors, COX-2, iNOS, (3) regulate cell detachment via down regulation of adhesion molecules such as cadherins, and (4) induce cell migration and invasion through matrix degrading enzymes [57–59] (Table 1, Fig. 1).

#### Resistance to chemotherapy and radiotherapy mediated by HIF1 signalling

Drug resistance could potentially occur at the cellular level or secondary to changes in the tumour microenvironment. Tumours have convoluted vasculature which results in proliferating well-nourished cells closer to the functional blood vessels and regions of hypoxic cells located away from the functional blood vessels. Irregular blood flow and large distances between functional blood vessels in solid tumours lead to poor drug distribution, resulting in therapeutic resistance [60].

It is often difficult to discriminate between the effects of hypoxia per se and HIF1, and the literature on the effects of the hypoxic microenvironment and HIF1 on drug efflux and multidrug resistant phenotype [61], for instance, is controversial. Zhao and co-workers recently reported that HIF1alpha suppresses MDR1/P-glycoprotein in gastric cancer by inhibiting miR-27a expression in gastric cancer [62], and in colon cancer cells, inhibition of HIF1 leads to downregulation of *p*-glycoprotein and reversal of multidrug resistant phenotype [63]. In contrast, pronounced hypoxia has minor effect on *p*-glycoprotein expression and activity [64], while acidosis, a feature of the hpoxic micro-environement, increases *p*-glycoprotein activity [65]. In addition, regarding drug efficacy, the hypoxic environment can modify the efficacy of drugs that require molecular oxygen as part of their mechanism of action, e.g., bleomycins [66, 67], or are activated by reductases under hypoxia, e.g., evofosfamide, tarloxotinib, tirapazamine, and SN30000 [68, 69], and is a barrier to drug delivery generally independent of HIF1 [70]. Hypoxia and HIF1 also confer treatment resistance of cancer cells by inducing cell cycle arrest (quiescence) [71], making drugs that target cycling cells ineffective), and by supporting the highly tumourigenic stem cell niche [72]. In glioblastoma, HIF1alpha+ quiescent stem-like are found to locate within the peri-necrotic region and confer higher tumourigenic potential [73]. During severe or prolonged hypoxia, most of the cells undergo programmed cell death. However, some of the tumour cells adjust to environmental stress and survive by avoiding necrosis, inhibition of apoptosis [48, 74, 75], and decreasing senescence of cells [76], mediated by HIFalpha, resulting in an aggressive phenotype and resistance to treatment.

During fractionated radiotherapy, HIF1alpha protects the tumour microvasculature from radiation-induced endothelial apoptosis, via induction of vascular endothelial growth factor (VEGF) and other pro-angiogenic factors and facilitates tumour cell survival by increasing the antioxidant capacity of tumours to counteract radation-induced oxidative stress [21]. Irradiation also induces changes in the tumour microenvironment such as vascular, stromal, and immunological changes which may promote radioresistance and tumour recurrence [77]. These effects eventually lead to the resistance of tumour cells to chemotherapy and radiation.

### Measurement of hypoxia

Knowledge of the hypoxia state enables prediction of treatment outcome and selection of patients for hypoxia modifying treatment. The relative prevalence of diffusion limited hypoxia (DLH), cyclic and perfusional hypoxia in human tumours or animal models is not known, and it is predicted that different hypoxia modes require different diagnostic and therapeutic approaches. Several noninvasive techniques (direct or indirect measurements) are available to obtain an absolute or relative value of the oxygenation status of tumours. The various strategies available are described in Table 2 [78–103]. Each of the techniques described interrogates different aspects of the hypoxic microenvironment, and they provide information on hypoxia at different locations: Oxygen electrodes and OxyLite sampling predominantly measure interstitial hypoxia. PET, SPECT, and extrinsic markers report on intracellular hypoxia and PET/SPECT images quantify data on a macroscopic scale in tumour regions. Blood oxygen level-dependent (BOLD)-MRI and Oxy-R fraction allow assessment of blood oxygenation, while indirect methods that report on hypoxia-induced molecular events (e.g., GLUT1, CA9 expression) rather than hypoxia itself have also been utilised as markers of tumour oxygenation.

**Table 2:** Methods of hypoxia assessment

Technique	Oxygen sensing range (pO_2_)	Mechanism	Advantages	Disadvantages
Direct measurements				
*Oxygen electrodes *(Eppendorf probes) [77–82]	0–100 mmHg	Allows direct point measurements of the partial oxygen pressure A polarographic needle is inserted in a tumour and several measurements along a track are obtained	Strong correlation with clinical outcome in patients with HN, cervical, or prostate cancer	Invasive, technically demanding Suitable only for accessible tumours Results are operator dependent No longer commercially available Risks modifying the oxygen concentration
*OxyLite* (fibre optic technique) [84]		Allows continuous measurement at a single spot in a tumour, whereby changes in oxygenation within a short period of time (several hours) can be obtained	Promising preclinical studies	Invasive Never approved for clinical use
Indirect measurements				
*Exogenous biomarkers* Bioreductive Nitroimidazole drugs Pimonidazole [85] and EF5 [86] Fluorescent Pimonidazole [87, 88]	<1 mmHg	Bioreduction and trapping under hypoxia. Predominantly derivatives of nitroimidazole compounds These can be chemically bound to an immune-reactive side chain, or fluorescent marker, for IHC detection of hypoxia Estimates the distance from cells to a vessel (radioresistant cells are further away from vessels)	Can be used with biopsies or surgical specimens Objective quantitation of hypoxia using immunohistochemistry or flow cytometry or fluorescent probes Provides information about the microregional distribution of hypoxia Indicate biologically relevant hypoxia because of its radioresistance	Invasive (requires biopsy or resection) Drug administered in advance Binding of drug dependent on the presence of tissue reductases Careful tumour sampling necessary to account for heterogeneity
*Endogenous biomarkers* [89–97]	<10 mmHg	Consist of proteins that are predominantly under control of HIF: HIF1, HIF2, CA9, GLUT-1, and vascular endothelial growth factor (VEGF) High expression of these is correlated with treatment failure in various cancers	Can be used with archival biopsies or surgical specimens without the need for prior drug administration Provides information about the microregional distribution of hypoxia	Invasive (requires biopsy or resection) Gene and protein expression influenced by hypoxia- independent factors Careful tumour sampling necessary to account for heterogeneity
* MRI* Blood oxygen-dependent level (BOLD) and tissue oxygen level-dependent (TOLD) magnetic resonance imaging (MRI) [102]	Poor correlation with absolution pO_2_ levels(BOLD)	BOLD: paramagnetic deoxyhemoglobin molecules in red blood cells cause magnetic susceptibility, which increases the local transverse MRI relaxation rate (R2; units ms^−1^)	Noninvasive Assessment of the entire tumour volume Spatial mapping of hypoxia Serial assessment over time Better spatial and temporal resolution of TH than PET provides	Indirect measure of hypoxia (deoxyhemoglobin concentration) Strong dependence on perfusion Susceptible to motion artefacts (BOLD), or absolute value is influenced by adequacy of oxygen saturation during inhalation (Oxy-R fraction)
*Oxy-R fraction* derived from oxygen enhanced MRI [98]	Probably 0–100 mmHg	Oxy-R fraction: dissolved oxygen in blood plasma and/or interstitial space when hyperoxic gas is breathed increases longitudinal MRI relaxation rate (R1; units s^−1^) in well perfused tissue	Quantifies the spatial distribution and extent of tumour oxygen delivery in vivo Can be readily quantified on clinical MRI scanners	Awaiting oncology clinical translation
* MR Oximetry* Based on perfluorocarbons (PFCs) [99–101]	Probably 0–100 mmHg	Sequestered in the reticuloendothelial system (liver, spleen, bone marrow) due to macrophage accumulation	Correlates with pO2 Long half-life enables chronic hypoxia evaluation	Susceptible to flow artefacts Doses for imaging causes hepatosplenomegaly
*PET* 2-Nitroimidazole [^18^F]labelled tracers (MISO, FMISO, EF5, FAZA, and HX4) Other tracers ([^60^Cu]Cu-ATSM) [103]	<1 mmHg	Redox-based trapping	Noninvasive Assessment of the entire tumour volume Spatial mapping of hypoxia Serial assessment over time	No consensus about preferred tracer False-positive results from unbound tracer Uptake in bladder and other normal tissues Limited spatial resolution Low tumour-background ratio

### MRI methods

These include MR-based gradient-recalled echo techniques, electron paramagnetic resonance, and MR spectroscopy. MRI methods for interrogating tumour oxygenation are attractive since MRI scanners are widely available and they avoid the complication of short-lived radioactivity.

#### Blood oxygen level dependent (BOLD)-MRI

The most facile contrast mechanism, which depends on blood oxygenation—blood oxygen level dependent (BOLD) MRI—avoids the need for reporter molecules by imaging differences between diamagnetic oxy-haemoglobin and paramagnetic deoxy-haemoglobin. The presence of deoxy-haemoglobin in a blood vessel causes susceptibility differences between vessel and its surrounding tissue resulting in a decrease in T2* leading to darkening in tissues containing the vessel in a T2*-weighted imaging protocol. A limitation is that it is also sensitive to changes in Hb concentration (due to alterations in vascular volume and flow as well as interconversion of oxy- and deoxy-haemoglobin). Therefore, this technique provides qualitative assessment of changes in oxygenation rather than quantitative measurements. The technique is widely used for functional brain mapping [104, 105], where it is thought to primarily reflect changes in flow.

Baudelet and Gallez have rigorously investigated correlations between pO_2_ estimated using fibre optic probes and BOLD signal changes and have found general correlations, but a given BOLD response may reflect vastly different changes in pO_2_ [102]. BOLD MRI has the advantage of both high spatial and temporal resolution and it can be repeated as needed; however, it can be susceptible to subtle motion artefacts [106]. Rijpkema et al. used BOLD to evaluate patients during the ARCON trial for head and neck cancer and found significant changes in T2*-weighted MRI contrast accompanying hyperoxic gas breathing [107]. No accompanying changes were observed by traditional T1-weighted gadolinium dynamic contrast-enhanced MRI. Preliminary analysis of 11 women being treated with chemotherapy for locally advanced breast cancer showed a significantly different BOLD response to breathing oxygen before the course of chemotherapy for tumours of women with good therapeutic outcome versus those with poor response. Indeed, three women with complete pathologic response showed a signal change greater than 7%, whereas those with poor outcome showed less than 3% [108]. It is arguable whether the differential response reflects perfusion or oxygenation, but traditional dynamic contrast-enhanced MRI failed to provide similar discrimination.

The biologic sequelae of hypoxia are also amenable to imaging. Prolonged hypoxia can lead to increased lactate in tissues and ^1^H MRI can be used to image lactate [109, 110]. Furthermore, alteration of the redox state of nonprotein thiols, such as glutathione, adenine nucleotide redox state, NADH or NADPH in hypoxic cells can lead to accumulation of radiopharmaceuticals in hypoxia. All of these tests measure downstream consequences of hypoxia and often do not instantly return to normal values after an adequate O_2_ supply has been established. For more information, the reader is referred to a recent review that addressed the role of functional MRI (fMRI) methods to assess tumour oxygenation for predicting outcome [111].

### PET imaging of hypoxia

Positron emission tomography (PET) has inherent advantages for studying hypoxia, as it can employ radiotracer probes that directly report on cellular oxygen levels, and not via hypoxia-mediated changes in phenotype, thereby permitting the non-invasive and three-dimensional assessment of intratumour oxygen levels in a more direct manner [112]. In contrast to histologic characterisation, PET can monitor whole tumours although at low spatial resolution [113]. PET has very high sensitivity and specificity compared to MR imaging and it enables the identification of regional hypoxia in vivo in preclinical and clinical settings [103].

#### PET tracers for hypoxia imaging and their mechanisms of action

The criteria for development of radiotracer probes includes improving relative tumour uptake by using isotopes with longer half-lives and ensuring rapid clearance of the parent compound from systemic circulation and normoxic tissue (hydrophilic compounds), while being sufficiently lipophilic to enter cells and allow uniform tissue distribution. The charcteristics of an ideal hypoxic tracer include: retention in low partial oxygen pressure (pO_2_) regions (hypoxia specific), pharmacokinetic profile and tissue distribution independent of confounding factors such as blood flow/tissue perfusion or pH, high stability, suitable tissue kinetics to enable imaging in a specified time frame, ease of synthesis, favourable dosimetry profile, reproducibility and effectiveness in multiple tumour types.

Radionuclide detection of hypoxia in tumours was first reported in 1981 with [^14^C]misonidazole autoradiography [114]. Subsequently, two main tracer classes have been developed to specifically study regional tumour hypoxia with PET: [^18^F]labelled nitroimidazoles and Cu-labelled diacetyl-bis(N4-methylthiosemicarbazone) analogues [112]. Multiple PET tracers suitable for the detection of hypoxia have been developed, validated and shown to exhibit different characteristics; some of these are discussed in Table 3 [115–127]. The first [^18^F]labelled drug to be clinically tested was fluoromisonidazole (FMISO) [128] and it remains the most extensively tested agent [129, 130].

**Table 3:** Salient characteristics of hypoxic radiotracers

Class	Mechanism of action	Advantages	Limitations
Nitroimidazoles			
[^18^F]FMISO [115]	Nitroimidazole compounds are used for imaging oxygen-deprived hypoxic cells, based on the intracellular accumulation of radicals formed after the reduction by ubiquitous nitroreductases. Under oxygenated conditions, in contrast, the nitro radical anions of the compounds are reoxidized and cleared from cells by back-diffusion	Lipophilicity ensures facile cell-membrane penetration and diffusion into tissue FMISO uptake correlates with pimonidazole immunohistochemistry in various cancers	Only available for research purposes. Modest hypoxic-to-normoxic tissue ratios (due to limited clearance) and limited hypoxic contrast potentially impedes visual detection of hypoxic regions: limited diagnostic utility in routine practice Slow tracer accumulation and low tumour-to-background contrast requires delayed scans to allow background activity to decrease [116, 117]
[^18^F]FAZA [118]		More hydrophilic: faster clearance kinetics, resulting in improved hypoxia-to-normoxia contrast	Not widely available
[^18^F]EF5 [119]		Greater cell membrane permeability and slower blood clearance leads to improved rates of tumour uptake and homogeneity of tracer distribution	Complex labelling chemistry and slow elimination due to higher lipophilicity
[^18^F]HX4 [120]		Hydrophilic Shorter acquisition times	No advantage over FMISO
[^18^F]FETNIM [121, 122]		Rapid renal clearance and low liver uptake	No advantage over FMISO Not widely available
[^18^F]RP-170 [123]		Shorter acquisition times, Improved hypoxic contrast	Not widely available
SR4554 [124, 127]		MR spectroscopy method analogous to FMISO but requiring measurement of elimination kinetics	Not widely available
Copper-diacetyl- bis(N4-methylthiosemicarbazone) (Cu-ATSM)			
Cu-ATSM [124, 125]	The hypoxic specificity of Cu-ATSM is thought to be partly imparted by the intracellular reduction of Cu(II) to Cu(I). Under hypoxic conditions, the unstable Cu(I)-ATSM complex may further dissociate into Cu(I) and ATSM, leading to the intracellular trapping of the Cu(I) ion	Simpler synthesis/radiolabelling methodology Reveals ‘hypoxic’ tissue within 10-15 min after IV administration mainly due to its rapid tracer kinetics Cu-ATSM uptake might better represent a general prognosticator of poor treatment response	Limited availability of Cu isotopes, Only produced at a few research sites

### [^18^F]Nitroimidazoles

**Fig. 2:**
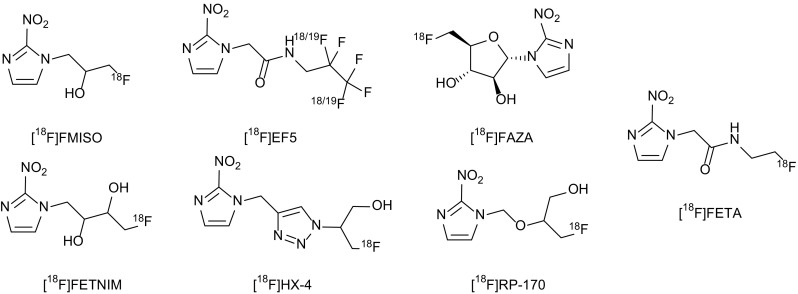
Structures of clinically used [^18^F]-labelled nitroimidazole compounds

**Fig. 3:**
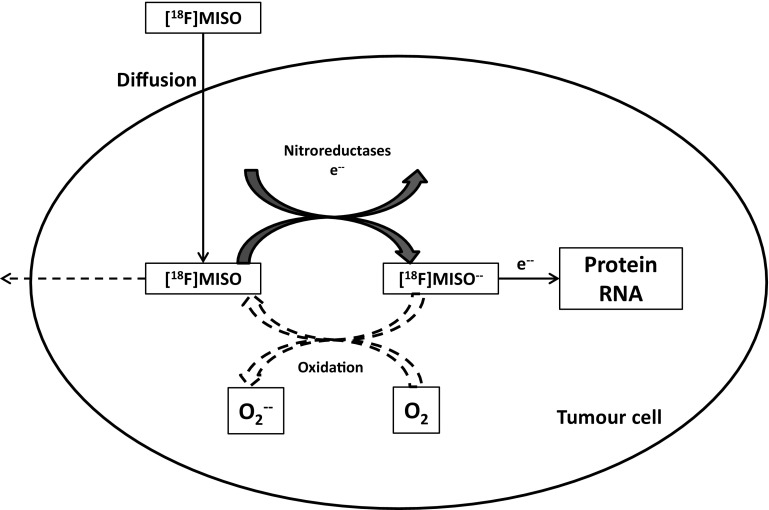
Schematic representation of FMISO uptake in hypoxic conditions

2-Nitroimidazole compounds were originally developed as hypoxic cell radiosensitisers and were introduced as hypoxia markers in the 1970s (Fig. 2) [115]. Nitroimidazoles enter cells by passive diffusion and subsequently undergo reduction forming reactive intermediate species. Hypoxic conditions cause further reduction of the nitro-anion radical, which is irreversibly trapped in the cell when the oxygen tension is less than 10 mmHg [129]. The reduction of nitroimidazoles requires the presence of ubiquitously expressed tissue reductases, which enables these compounds to accumulate within viable hypoxic cells, but not apoptotic or necrotic cells [130–132]. Under normoxic conditions, nitro-anion radical is re-oxidised into the parent compound, which can diffuse out of the cell. The mechanism of of [^18^F]MISO intracellular trapping is shown in Fig. 3 [133]. Therefore, hypoxic tissues can be delineated as an area of high tracer uptake after allowing a sufficient period of time for the nonspecific tracer to be excreted from the cells [134, 135].

FMISO uptake was closely correlated with pimonidazole immunohistochemistry and has been found to reflect hypoxia in head-and-neck cancer [136–146], glioma [147–152], colorectal cancer [153], breast cancer [154], lung cancer [155, 156], and renal cell carcinoma [157, 158].

**Fig. 4:**
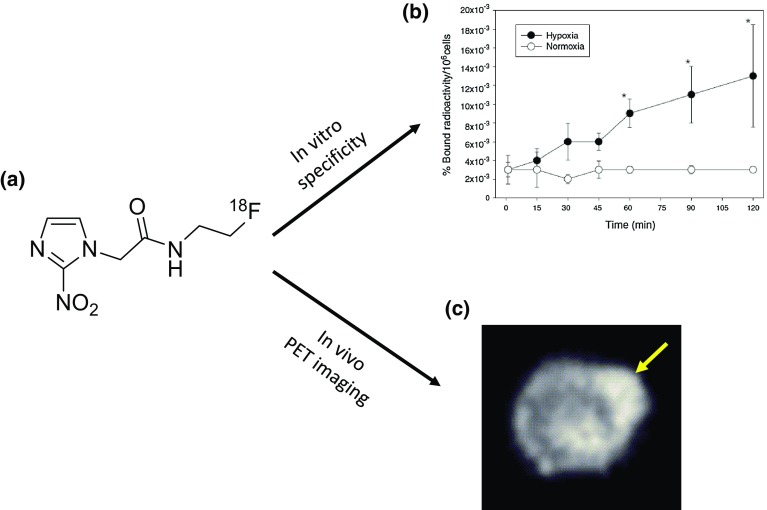
Hypoxia imaging with radiolabelled 2-nitroimidazole. **a** Chemical structure of [^18^F]fluoroetanidazole. The nitro moiety is necessary for hypoxia selective retention. **b** Cellular uptake of [^18^F]fluoroetanidazole in RIF-1 cell line culture grown under normoxia or hypoxia (nitrogen gas). The amount of radioactivity bound to cells was counted. **c** Imaging of [^18^F]fluoroetanidazole by PET showing tracer localisation in HT1080 (subclone 1-3C) xenograft. A 0.5-mm transverse slice of the 30–60 min image acquired in a small animal PET scanner is shown. *Arrow*, tumour. Courtesy of EOA Published in Br J Cancer 2004 (Barthel et al.) (Color figure online)

In view of FMISO’s slow plasma clearance, FMISO imaging usually requires an interval of longer than 2 h (ideally 4 h) after administration to obtain good contrast [159] with a hypoxia threshold in general defined as SUVmax of 1.5 or tumour:muscle ratio of 1.4 [103]. Although its biodistribution properties do not result in high-contrast images, the 2-h image unambiguously reflects regional pO_2_ in the range where it is clinically significant. However, due to perceived concerns regarding FMISO stability in vivo [160], metabolite formation, slow clearance properties [129], and failure to achieve image intensities in humans comparable to what had been achieved in animal models, alternative hypoxia PET tracers with different clearance and hydrophilicity characteristics have been developed in an attempt to overcome these limitations. These include fluoroazomycin arabinoside (FAZA), fluoroerythronitroimidazole (FETNIM), fluoroetanidazole (Fig. 4), and fluorinated etanidazole derivatives (EF3, EF5), HX4 [161–163].

### [^18^F]Fluoroazomycin-arabinofuranoside (FAZA)

[^18^F]Fluoroazomycin-arabinofuranoside (FAZA) is more hydrophilic than FMISO. Consequently, it has faster clearance kinetics, resulting in improved tumour-to-reference tissue ratios, and thus hypoxia-to-normoxia contrast. Head-to-head comparisons between FAZA, [^124^I]IAZA, and FMISO in preclinical animal studies imaged at 3 h after injection demonstrated faster vascular clearance of FAZA, resulting in an increased tumour-to-blood ratio (5.19) relative to that of [^18^F]fluoromisonidazole (3.98). More recently, clinical studies have successfully evaluated the feasibility of FAZA for imaging hypoxia in gliomas [118], lymphomas [118], lung [164, 165], head-and-neck [118, 166–168], cervical [169], and rectal tumours [170], and the results have been shown to compare favourably with equivalent FMISO data, especially as improved hypoxic–normoxic contrast was obtained at earlier time points. High FAZA tumour-to-reference tissue values have been associated with reduced disease-free survival and have shown prognostic potential in the detection of hypoxia in head-and-neck patients [167]. Due to the higher tumour-to-reference tissue ratios in comparison with FMISO, FAZA is gaining popularity for PET imaging of tumour hypoxia. Despite the fact that FAZA is not widely available at present, increasing research demand may persuade more sites to produce it.

### Next-generation tracers

#### [^18^F]2-(2-nitro-1H-imidazol-1-yl)-*N*-(2,2,3,3,3-Pentafluoropropyl)-acetamide (EF5)

The nitroimidazole EF5 has been extensively used for ex vivo immunohistochemical detection of bioreduced adducts, which indicate regions of tumour hypoxia. However, [^18^F]2-(2-nitro-1H-imidazol-1-yl)-N-(2,2,3,3,3-pentafluoropropyl)-acetamide (EF5), first investigated as a hypoxia PET tracer in 2001 [171], has only recently appeared in the clinical setting. In contrast to many of the second-generation hypoxia tracers, EF5 is highly lipophilic, resulting in greater cell membrane permeability and slower blood clearance [119], thus improving rates of tumour uptake and homogeneity of tracer distribution. The main drawback of EF5 is the complex labelling chemistry in comparison to the simple nucleophilic displacement reactions used for the mono-fluorinated 2-nitroimidazoles [171].

### [^18^F]3-fluoro-2-(4-((2-nitro-1H-imidazol-1-yl)Methyl)-1H-1,2,3-triazol-1-yl)propan-1-ol (HX4)

[^18^F]3-fluoro-2-(4-((2-nitro-1H-imidazol-1-yl)methyl)-1H-1,2,3-triazol-1-yl)propan-1-ol (HX4), a next-generation 2-nitroimidazole tracer contains a 1,2,3-anti-triazole moiety (as a synthetic convenience) rendering it more hydrophilic than FMISO, specifically designed to maximize pharmacokinetic and clearance properties. Initial studies in humans demonstrate rapid renal clearance and urinary excretion of HX4, with a favourable dosimetry profile similar to that of FMISO [120].

Preclinical studies validated that the tracer uptake was indeed oxygen-dependent though tumour-to-background ratios appeared similar to those reported for FMISO in studies using the same tumour model [172]; thus, it remains to be seen if HX4 provides any significant advantage over FMISO in a clinical setting. A phase I study of 6 patients (4 non-small-cell lung carcinoma, 1 thymus carcinoma, and 1 colon carcinoma) has shown a median tumour-to-muscle ratio of 1.40 at 120 min after injection, although no attempt was made to determine the optimal imaging time points [120]. In head-and-neck tumours, HX4 produced tumour-to-reference tissue values similar to FMISO at relatively early time points post injection, indicating the potential advantage of shorter acquisition times [173]. A more recent study in non-small cell lung cancer (NSCLC) patients [174] suggested that later scan times (2–4 h p.i.) can further enhance the hypoxic-to-normoxic signal.

#### [^18^F]Fluoroerythronitromidazole (FETNIM)

The hydrophilic nature of [^18^F]Fluoroerythronitromidazole (FETNIM) accounts for its rapid renal clearance and low liver uptake, compared with FMISO. This also could explain the positive correlation between tumour blood flow and initial tumour FETNIM uptake [121]. Recent clinical studies in head-and-neck [121, 175, 176], lung [177, 178], cervical cancer [179], and oesophageal cancer [180] showed that high tissue uptake of FETNIM was indicative of reduced progression-free and overall survival. However, as with HX4, it is not clear whether the use of this tracer presents any advantages over FMISO imaging protocols. Clinical studies with FETNIM have been mainly carried out at the University of Turku, Finland.

### [^18^F]1-(2-1-(1H-methyl)ethoxy)-methyl-2-nitroimidazole (RP-170)

1-(2-1-(1H-methyl)ethoxy)-methyl-2-nitroimidazole was developed as a 2-nitroimidazole-based hypoxic radiosensitiser, which has also been labelled with fluorine-18 ([^18^F]RP-170). The hypoxic selectivity of RP-170 was demonstrated in glioma patients on the basis of significant correlations between uptake, oxygen tension measurements, and HIF1alpha immunostaining [123]. Studies in brain [123, 181] and lung [182] tumours indicated higher SUV (calculated at 1 h post injection), for hypoxic than normal tissues. The shorter interval before scanning, combined with improved hypoxic contrast compared with FMISO, could make it attractive for clinical imaging.

### Copper (Cu)-diacetyl-bis (N4-methylthiosemicarbazone) (Cu-ATSM)

**Fig. 5:**
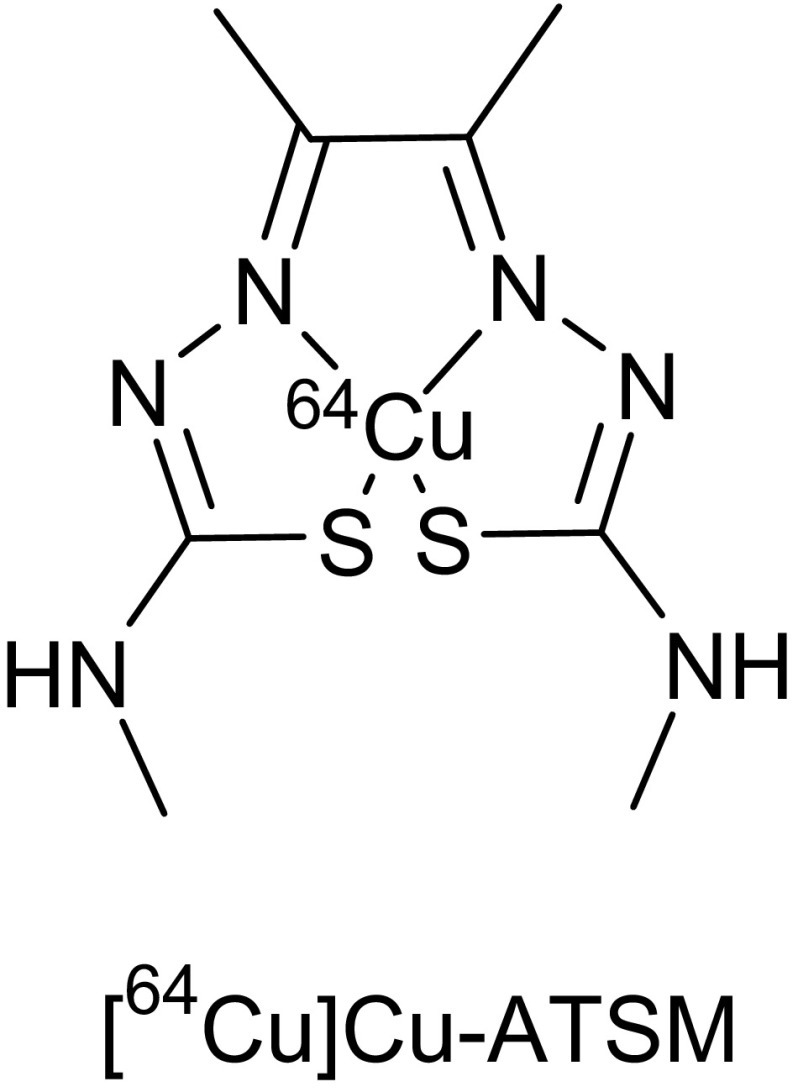
Structure of [^64^Cu]Cu-ATSM

**Fig. 6:**
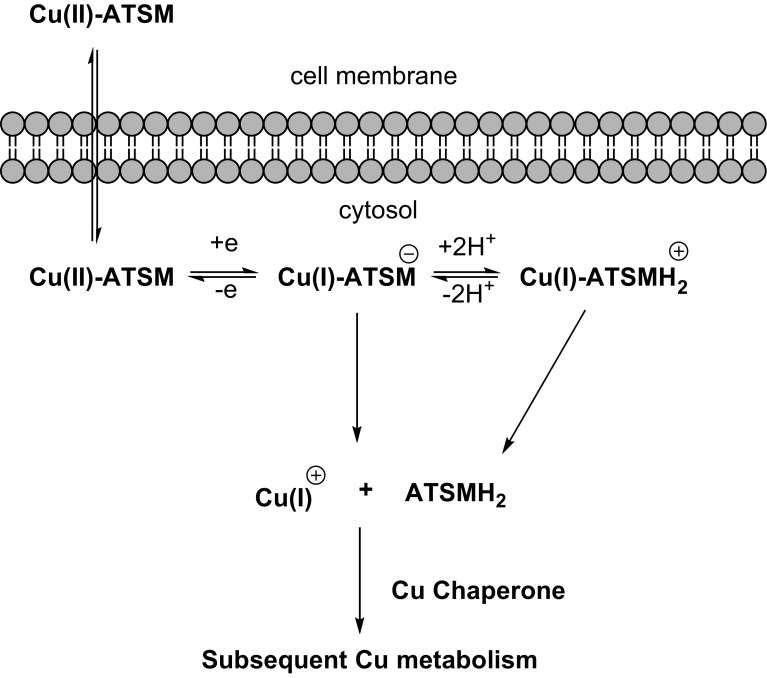
Schematic representation of proposed mechanism of [^64^Cu]Cu-ATSM

An alternative class of agents for the study of hypoxia with PET that has been intensively investigated in both preclinical and clinical studies is the complex of Cu with diacetyl-bis(N4-methylthiosemicarbazone) (ATSM) ligands, among which ATSM is the prototype (Fig. 5). The potential of these agents for hypoxia imaging was first reported by Fujibayashi et al. [124]. Copper (Cu)-diacetyl-bis (N4-methylthiosemicarbazone) (Cu-ATSM) is a hypoxic marker that is selectively retained in hypoxic tissues. Cu-ATSM rapidly diffuses into the cells due to its high membrane permeability and low redox potential, secondary to its lipophilicity and low molecular weight. After cellular entry, Cu(II)-ATSM is reduced to an unstable Cu(I)-ATSM species, which further dissociates into the metal complex Cu(I), and ATSM, thus irreversibly trapping the Cu(I) within the cellular copper metabolic processes (Fig. 6) [183]. In normoxic conditions, the [Cu(I)-ATSM] can be re-oxidised to its parent compound, allowing efflux from the cell [184]. One of the advantages of Cu-ATSM is that it can reveal molecular contrast within 10–15 min post injection mainly due to its rapid tracer kinetics [125, 185].

However, it has been observed that high uptake in tumours may only partly be a direct consequence of hypoxia [185]. Nevertheless, extremely high-contrast images of Cu-ATSM have been obtained in a variety of tumour sites [186]. The lack of correlation between Cu-ATSM distribution and immunohistochemistry hypoxia markers casted some doubt on the hypoxia selectivity of Cu-ATSM [187]. The suggested reason for the low correlation between Cu-ATSM uptake and hypoxic distribution, in some tumours, was the differing redox status of the tumour types. This has been further seen in pre-clinical experiments, where it was demonstrated that in the cell lines tested, [^64^Cu]Cu-ATSM and [^64^Cu]Cu-acetate had almost identical uptake in vivo over 2–16 h, post injection. However, up to 1 h post injection, [^64^Cu]Cu-acetate had a superior tumour-to-muscle ratio [188]. Several factors could explain the phenomenon; indeed, some tumours might have a lower than-average redox potential with high concentrations of electron donors causing reduction and trapping of Cu-ATSM in both hypoxic and normoxic areas. This observation does not discount the fact that [^64^Cu]Cu-ATSM may still be clinically relevant as a tracer for hypoxia, perhaps HIF1 status, as suggested by some investigators [189]. The timing of image acquisition is crucial, as the initial phase of tracer uptake can be perfusion and hypoxia-driven, whereas at later time points uptake is probably more indicative of tumour hypoxia.

### Validation of MRI and PET hypoxia imaging

As discussed thus far, both MRI and PET play an important role in hypoxia imaging. However, there are few reports that compare these two imaging modalities. Preclinically, a clear correlation between [^18^F]FAZA PET image intensities and tumour oxygenation was demonstrated by Tran et al. [190]. [^18^F]FAZA accurately showed improved uptake when rats with subcutaneous rhabdomyosarcomas were treated with air, in contrast to carbogen. This correlated well with an invasive OxyLite probe, although the probe demonstrated a relatively high heterogeneity in the oxygen value measured depending on the specific point within the tumour. Functional MRI ([^19^F]MRI, however, did not show any discernible difference in T1 spin–lattice relaxation time. In a more recent study, Valable et al. validated tissue saturation studied by MRI against FMISO PET wth high sensitivity and specificity in a rat glioma model [191].

In the clinical setting, Swanson et al. performed a detailed spatial analysis of the hypoxic tumour burden visible on the FMISO PET relative to the imaging changes associated with tumour neovasculature, necrosis, invasion, and edema seen on gadolinium-enhanced T1-weighted MRI (T1Gd) in 24 patients with glioblastoma [152]. Hypoxic Volume (HV), defined within the tumour as sections that had a tumour-to-blood ratio of higher than 1.2, showed a consistent correlation with the MRI-defined regions within the tumour, supporting the idea that there is a definite link between the PET and MRI images of hypoxia. Furthermore, it was found that HV, and the respective surface areas of HV and T1Gd abnormality were the most significant predictors of survival. Simoncic et al., showed a strong correlation between FMISO PET and dynamic contrast enhanced MRI (DCE-MRI) kinetic parameters in 6 head and neck cancer patients [192].

These studies suggest that both MRI and PET could complement each other and provide a future direction in selecting the best modality to image hypoxia.

### Clinical applications

There is evidence from numerous clinical studies across a range of tumour types to support the existence and importance of the “hypoxia driver” phenotype both in pre-clinical [193–219] (Table 4) and clinical studies [220–247] (Table 5).

**Table 4:** Preclinical studies of hypoxia imaging

FMISO	
Rasey et al. [213]	Uptake of FMISO by V79 multicellular spheroids after 4 h of incubation with [^3^H]FMISO, provided a visual and a quantitative measure of hypoxia. Autoradiographs revealed heavily labelled cells in an intermediate zone between the well-oxygenated periphery and the necrotic center
Martin et al. [205]	Evaluated the relationship between oxygen concentration and [^3^H]FMISO binding in monolayer preparations of isolated adult rat myocytes. Under anoxic conditions, [^3^H]FMISO binding after 3 h was approximately 25-fold greater than normoxic controls, which reduced to 40% at a pO2 of 4 mmHg. [^3^H]FMISO uptake was independent of glucose or thiol concentrations, cellular pH, potential confounding variables in the tumour microenvironment
Martin et al. [204]	Confirmed that FMISO uptake was independent of blood flow, both in individual tumours and normal tissues
Troost et al. [216]	FMISO can be used to monitor treatment-induced changes in tumour hypoxia, similar to that seen with pimonidazole in various tumour models
Troost et al. [138]	Established a correlation between pimonidazole staining and FMISO distribution in head-and-neck xenografts. FMISO accumulation was dependent on the presence of hypoxia and on the tumour microarchitecture
Oehler et al. [153]	It is feasible to distinguish between different tumor responses to DMXAA treatment. A reduction in FMISO uptake was related to reduced perfusion and, therefore, delivery of FMISO, rather than a reduction in tumour hypoxia
Murakami et al. [157]	Early changes in the tumour microenvironment following anti-angiogenic therapy confirmed tumour starvation with FMISO hypoxia imaging
Hatano et al. [147]	Intratumoral FMISO distribution reflected tumor hypoxia and expression of the hypoxia related gene product GLUT-1. However, it did not reflect tumor proliferation or glucose metabolism
Schutze et al. [219]	Showed that pretreatment FMISO hypoxic volume in FaDu hSCC xenografts is prognostic. SUVmax was not associated with local control
FAZA	
Sorger et al. [215]	In vitro and invivo study in rat carcinosarcoma tumour models comparing FMISO with FAZA, demonstrated similar tracer accumulation in sites of hypoxia on early PET imaging. However, FAZA had faster elimination kinetics and was cleared via the renal system
Piert et al. [212]	Confirmed the faster clearance of FAZA in murine mammary carcinoma, squamous cell carcinoma, and pancreatic acinar cell carcinoma. FAZA had a lower tumour/blood ratio compared with FMISO
Chapman et al. [196]	Sunitinib treatment resulted in improved tumour oxygenation as shown by significant reduction in FAZA uptake in Caki-1 renal cell xenografts. FAZA uptake increased again upon sunitinib withdrawal, indicating a rebound in tumour hypoxia
Chang et al. [195]	Demonstrated the feasibility of FAZA PET as an early pharmacodynamic monitor on the efficacy of anticancer agent BAY 87-2243 that targets the mitochondrial complex I and intratumour oxygen levels
EF5	
Chitneni et al. [197]	Demonstrated the utility of EF5 PET for monitoring early response to tumour treatment with SN30000 (a novel hypoxia-activated prodrug) plus RT in H640 non-small cell lung cancer xenografts
Silvoniemi et al. [215]	In their evaluation of the relationship between hypoxia (evaluated with EF5 PET) and tumour growth, have demonstrated that uptake of EF5 in the late phase of exponential tumor growth is associated with the tumour growth rate in mice bearing HNC xenografts
Chitneni et al. [198]	Evaluated EF5 tumour uptake versus EF5 binding and hypoxia as determined from immunohistochemistry at both macroscopic and microregional levels. It was shown that the uptake and hypoxia selectivity of [^18^F]EF5 varied among tumour models-PC3, HCT116, and H460
Ali et al. [193]	Evaluated the relationship between pre-treatment EF5 PET and the response of preclinical tumor models (HT29, A549 and RKO tumours grown in nude mice) to a range of fractionated radiotherapies. Irradiated tumours exhibited reduced EF5 uptake 1 month after treatment compared to control tumours, suggesting that pre- treatment EF5 PET can predict the response of tumours to single fraction radiation treatment
HX4	
Dubois et al. [200]	In a rhabdomyosarcoma rat tumour model, HX4 binding was dependent on tumoural oxygenation status. A significant spatial relationship was shown between HX4 distribution and pimonidazole staining
Carlin et al. [187]	In a SQ20b head and neck xenograft mouse model similar tumour to muscle ratios for FMISO, FAZA, and HX4 were seen. The fluorinated nitroimidazoles all showed radiotracer uptake increasing with pimonidazole and CA9 staining. However, (64)Cu-ATSM showed and inverse relationship. However, these results were obtained at 80–90 min post injection, a time point which is probably too early for evaluation since normal tissue clearance is still ongoing. Cu-ATSM had the highest tumour accumulation and low renal clearance compared to fluorinated nitroimidazoles
Peeters et al. [211]	In a comparative study within a rat rhabdomyosarcoma model, FMISO, FAZA, and HX4 uptake (tumour to blood ratio (TBR)), reproducibility and reversibility were assessed. Blood clearance for FAZA and HX4 was similar 3 h p.i., while for FMISO, as expected, clearance from normal tissues was significantly lower. Differences in tumour uptake resulted in significantly higher TBR for HX4 compared to the other tracers. Reproducibility was similar for both FMISO and HX4. Furthermore, decreasing the hypoxic fraction using carbogen resulted in loss of FMISO uptake, whilst modifying the hypoxic fraction by breathing 7% oxygen further enhanced FAZA and HX4 uptake
Peeters et al. [210]	Evaluated the efficacy of the hypoxia-activated cytotoxic prodrug TH-302. The hypoxic fraction assessed with HX4 PET imaging in the rhabdomyosarcoma model was significantly reduced at day 4 upon TH-302 treatment, while vehicle treatment was ineffective
FETNIM	
Gronroos et al. [201]	Comparison of FMISO and FETNIM uptake in C3H mammary carcinoma mice model demonstrated equivalence of both the tracers in terms of tumour oxygenation status and intratumoural uptake
Cu-ATSM	
Fujibayashi et al. [124]	Demonstrated that [^62^Cu]Cu-ATSM is reduced and retained in hypoxic tissues, whereas it rapidly washes out of normoxic tissues
Lewis et al. [203]	One of the first [^64^Cu]Cu-ATSM preclinical study in tumour hypoxia imaging in mice bearing EMT6 breast carcinoma cell line, has shown an heterogeneous uptake of the radiotracer (intense uptake was observed in 15–30% of the tumour)
Ko et al. [202], Obata et al. [209]	In an epidermoid rabbit tumour (with a high glycolytic/high hexokinase rate) accumulation of [^64^Cu]Cu-ATSM was seen around the outer rim of the tumour masses which on histology correlated with active, viable, and expected hypoxic cells
O’Donoghue et al. [185]	A good correlation of the intratumour distribution of Cu-ATSM and FMISO was seen at later imaging time points in a FaDu squamous carcinoma model but not at early time points in an R3327-AT anaplastic rat prostate tumour model. This is consistent with the hypothesis that the spatial distribution of FMISO and [64Cu]Cu-ATSM at later times reflects tumour hypoxia
Burgman et al. [194]	A similar study indicated that for early images, the distribution of Cu-ATSM was inconsistent with tumour hypoxia and might be more representative of perfusion. Correlation of Cu-ATSM and FMISO uptake at later time points was confirmed. The authors did not dispute the potential utility of Cu-ATSM imaging as a tool, but they pointed out that the mechanism of uptake in hypoxic tumour was unclear
McQuade et al. [208]	Demonstrated that tumour uptake of hypoxia-selective Cu-ATSM analogues (Cu-ATSE) decreases with increased oxygenation
Yuan et al. [218]	[^64^Cu]Cu-ATSM was shown to be a valid PET hypoxia marker (correlation of the autoradiographic distributions with hypoxia markers as EF5, pimonidazole, and CA9) for adenocarcinoma and glioma tumour cell line, but not in the fibrosarcoma model, where a hypoxia-independent uptake of [^64^Cu]Cu-ATSM was observed
Matsumoto et al. [206]	In a direct comparison involving Cu-ATSM, FMISO and pimonidazole in the SCCVII tumour model, uptake of both FMISO and pimonidazole decreased as oxygenation increased, as would be expected for a hypoxia imaging agent, but uptake of Cu-ATSM increased under identical conditions
Dence et al. [199]	The affinity of [^64^Cu]Cu-ATSM for viable and hypoxic cells was confirmed with the comparison of the regional distribution between [^64^Cu]Cu-ATSM and FMISO, FLT, and FDG. A very strong correlation of Cu-ATSM uptake with classical hypoxia (FMISO) and proliferation (FLT) was seen but there was no correlation with metabolic activity (FDG)
McCall et al. [207]	Confirmed a rapid tumour uptake and retention of [^64^Cu]Cu-ATSM (tumour-to-muscle ratio was 4:1 within 20 min after injection) with a strong positive spatial correlation to the highly perfused areas. At later time points (18 h post injection), the tumour-to-muscle ratio was 12:1 and there was no spatial correlation with the perfused areas
Valtorta et al. [217]	Evaluated the kinetics of [^64^Cu]Cu-ATSM distribution using [^18^F]FAZA as the gold standard in different xenograft mouse models (FaDu, EMT-6, and PC-3). Cu-ATSM showed a higher tumor-to-muscle ratio than FAZA with overlapping radioactivity distribution profiles in the FaDu mouse model, but heterogeneous distribution in EMT-6 and PC-3 models. This study confirmed the cell-dependent distribution and retention kinetics of Cu-ATSM and underlined the need for proper validation of animal models and PET acquisition protocols before exploration of any new clinical applications
Hueting et al. [188]	Demonstrated that the distribution of radiocopper from Cu-ATSM in tumours essentially mirrors Cu-acetate suggesting that copper metabolism might play a role in the mechanism of selectivity of Cu-ATSM

**Table 5:** Clinical hypoxia PET imaging studies in various tumours

Brain tumours	
FMISO	
Valk et al. [148]	The first clinical FMISO feasibility study in high-grade glioma involved 3 patients, where initial FMISO uptake in tumours was found to be greater than in normal cerebral cortex. Concurrent Rubidium-82 imaging showed blood brain barrier (BBB) defect at tumour site
Bruelhmeier et al. [149]	Evaluation of tumour perfusion with [^15^O]H_2_O PET scan in 11 patients with residual or recurrent brain tumour has shown that early FMISO uptake correlated with perfusion, but late FMISO uptake was independent of perfusion. Late FMISO PET provides a spatial description of hypoxia independent of BBB disruption and tumour perfusion
Cher et al. [151]	In 17 patients with malignant glioma, preoperative FMISO scans have been shown to be an accurate noninvasive marker of hypoxia (significant correlation between FDG and FMISO uptake with Ki-67 and VEGFR-1 expression), with FMISO uptake seen in all high-grade gliomas, and was prognostic for treatment outcomes
Swanson et al. [152]	In 24 patients with high-grade gliomas, the distribution of hypoxia seen on FMISO correlated spatially and quantitatively with the amount of leaky neovasculature seen on T1 weighted MRI images. The hypoxia volume generally straddled the outer edge of the T1 weighted MRI abnormality
FRP-170	
Shibahara et al. [181]	Imaging with FRP-170 PET aids visualisation of hypoxic lesions in 8 patients with glioma. SUVmax correlated positively with HIF–1a immunostaining
Beppu et al. [123]	Intratumoural pO2 measured using microelectrodes during tumour resection and HIFa immunostaining correlated with FRP-170 uptake in 12 patients with high-grade gliomas. The mean pO2 was significantly lower in the areas of high uptake than in those of low uptake, suggesting that high accumulation of FRP-170 might indicate viable hypoxic tissues
Cu-ATSM	
Hino-Shishikura et al. [225]	Tumour hypoxia assessed by [^62^Cu]Cu-ATSM PET/CT correlates with diffusion capacity obtained by diffusion weighted MRI imaging and may be useful for grading gliomas. [62Cu]Cu-ATSM uptake was significantly higher in high-grade gliomas than in normal or lower grade tumour tissues

Head and neck cancer (HNC)	
FMISO	
Gagel et al. [139]	A correlation was reported between the tumour-to-muscle ratio (TMR), the uptake of FMISO PET and pO_2_ polarography in 16 patients with HNC, indicating that FMISO-TMR is a suitable method for measuring tumour hypoxia. No correlation between tumour oxygenation status and FDG uptake was seen
Rajendran et al. [150]	FMISO imaging detected hypoxia in all tumour types, but there was poor correlation between glucose metabolism and hypoxia
Hicks et al. [141]	Positive FMISO uptake in 13 patients. Qualitative decrease in FMISO and FDG uptake induced by therapy
Thorwarth et al. [244]	Different types of hypoxia-perfusion patterns identified in tumours
Thorwarth et al. [142]	Preradiotherapy FMISO uptake was a prognostic indicator of treatment response to radiotherapy in 12 patients with HNC. There was no correlation between FDG and FMISO tumour uptake
Rajendran et al. [248]	In a study of 73 patients with HNC, pretreatment uptake of FMISO was found to be an independent prognostic factor and a stronger predictor of outcome. (Rajendran CCR 2006)
Rajendran et al. [237]	Evaluated the feasibility of FMISO guided radiotherapy boost in a patient with H and N cancer. This was feasible while respecting the organs at risk tolerance
Rischin et al. [249]	FMISO indicated the hypoxia status of each tumour. Higher risk of locoregional failure in hypoxic tumours, while patients on tirapazamine had lower risk of locoregional failure
Zimny et al. [143]	In metastatic HNC, FMISO retention is significantly greater in hypoxic tumours than in normoxic tumours, with a strong correlation between the FMISO uptake and Eppendorf pO2 histography readings of <5 mmHg. No correlation was found with FDG
Eschmann et al. [250]	Radiotherapy decreased FMISO tumour uptake
Gagel et al. [140]	Moderate correlation between oxygen measurements and FMISO uptake. Poor correlation between FDG and FMISO
Lee et al. [251]	Variable FMISO tumour distribution
Nehmeh et al. [235]	Good correlations of intratumour FMISO distributions was seen in 6/13 patients, (consistent with chronic hypoxia) when imaged 3 days apart in a reproducibility study
Dirix et al. [252]	Quantitative evaluations of FMISO uptake are expected to play an important role in dose escalation radiotherapy planning. Disease-free survival correlates negatively with baseline tracer uptake and initial hypoxic volume
Okamoto et al. [235]	Evaluated the reproducibility of FMISO uptake in HNC in 11 patients on two separate occasions 48 h apart. FMISO PET can identify hypoxic areas with high reproducibility, thus enabling accurate target delineation during radiotherapy planning
Lee et al. [252]	Heterogeneous distribution of FMISO was seen in the primary and/or nodal disease in majority of the patients
Jansen et al. [225]	Gadopentetate dimeglumine (Gd-DTPA)-based dynamic contrast-enhanced magnetic resonance imaging (DCE-MRI) was combined with FMISO PET in 13 node-positive HNC patients. FMISO uptake negatively correlated with tumour perfusion as assessed by DCE-MRI
Abolmaali et al. [145]	FMISO contrast increases 2–4 h post injection
Kikuchi et al. [254]	Disease-specific survival was significantly lower in patients with high baseline FMISO uptake
Yamane et al. [255]	FMISO tumour uptake and hypoxic volume significantly decreased after neo-adjuvant chemotherapy in 13 patients with HNC
Zips et al. [247]	Hypoxia PET imaging with FMISO after 1 or 2 weeks of radiotherapy correlated better with outcome than imaging pre-treatment
Sato et al. [146]	HIF1 expression was strongly correlated with FMISO uptake, but not with FDG uptake, suggesting that FMISO uptake in the primary site of oral squamous cell carcinoma (SCC) indicates a hypoxic environment with HIF1 expression
Tachibana et al. [243]	Showed that 9 of 10 patients with HNC had positive FMISO uptake before radiation therapy. A significant decrease in FMISO uptake was noted at 2 weeks of fractionated radiation therapy in all of the FMISO-positive tumours, indicating reoxygenation during radiotherapy
Sato et al. [240]	In a prospective study in 22 patients with oral SCC, FMISO, and FDG PET, done prior to neoadjuvant chemotherapy, demonstrated inverse relation between therapy response and FMISO uptake, whereas the FDG uptake was not significantly correlated with the chemotherapy response. Histological response was used as gold standard
FAZA	
Souvatzoglou et al. [168]	In 11 patients with HCN, FAZA uptake was seen in 7 of 11 primary tumours and 3 of 11 lymph node metastases. Physiological uptake in the kidney and hepatobiliary tree hampered diagnostic interpretation
Grosu et al. [166]	In patterns of uptake evaluation, FAZA uptake was seen as a single confluent region in 11/18 patients and as multiple diffuse regions in 4/18 patients
Postema et al. [118]	High tumour to blood ratio (TBR) in all 7 gliomas; high TBR, SUVmax observed in 6/9 H&N tumours; moderate TBR, SUVmax in 3/21 lymphomas; increased TBR, SUVmax in 7/11 lung patients
Mortensen et al. [167]	In a small series of 40 patients with HNC, pretreatment tumour hypoxic fraction measured using FAZA PET and an intensity threshold analysis technique was predictive of survival following radiotherapy. High uptake was associated with lower disease-free survival
Servagi-Vernat et al. [242]	Semi-quantitative assessment of hypoxic volume using FAZA PET before and during intensity-modulated radiation therapy (IMRT) for 12 patients with locally advanced HNC aids in the delineation of hypoxic volumes for dose escalation protocols
EF5	
Komar et al. [227]	EF5 PET could potentially be a surrogate marker of radioresistance. In 22 patients with HNC, high uptake of the hypoxia tracer EF5 showed a stronger correlation with a poor clinical outcome than FDG uptake
Maity et al. [233]	Ongoing trial at the University of Pennsylvanian evaluating reversal of hypoxia in HNC using nelfinavir which may assist the process of re-oxygenation that can occur with fractionated therapy. Patients will be evaluated using EF5 PET before and after the nelfinavir treatment, just prior to radiotherapy
HX4	
Chen et al. [173]	A comparative study in head and neck cancer patients found similar tumour to muscle ratios for HX4 imaging at 1.5 h p.i. and FMISO imaging at 2 h p.i
Zegers et al. [246]	In HNC, hypoxia PET imaging with HX4 provides complementary information to FDG imaging. On average 24% of the HX4 hypoxic volume was outside the FDG volume
FETNIM	
Lehtio et al. [121]	Uptake of FETNIM in HNC is highly variable and seems to be governed by blood flow at least in the early phase of tissue accumulation. Tumour distribution volume correlated strongly with FETNIM uptake and blood flow (measured by [15O]H_2_O), but not with FDG uptake. Values compare favourably with FMISO uptake
Lehtio et al. [176]	Tumour to plasma (T:P) ratio of FETNIM provided an estimate of tumour hypoxia in 10 patients with HNC
Lehtio et al. [174]	Patients with higher fractional hypoxic volumes and T:P ratio correlated with poorer survival
Gronroos et al. [224]	In 15 HNC patients treated with radiation, no correlation between FETNIM imaging results and endogenous expression of hypoxia markers such as HIF1 and GLUT-1 was found
Cu-ATSM	
Chao et al. [256]	Hypoxia imaging with 64Cu-ATSM guided IMRT dose escalation in a phantom study
Minagawa et al. [257]	All 5 patients with 64Cu-ATSM SUVmax < 5 were complete responders

Breast cancer	
FMISO	
Cheng et al. [154]	Showed that there is correlation between FMISO uptake and endocrine therapy outcome and poor correlation between FMISO uptake and HIF-1a immunostaining

Lung cancer	
FMISO	
Koh et al. [258]	In a study of 7 patients with non-small-cell lung cancer (NSCLC), no correlation between tumour size and fractional hypoxic volume, defined by FMISO PET, was observed. Radiotherapy reduced median fractional hypoxic volume from 58 to 22%
Eschmann et al. [259]	FMISO scans performed preradiotherapy in a group of 14 patients with NSCLC: a high TMR and tumour/mediastinal ratio was associated with a higher risk of relapse. FMISO imaging could identify postradiotherapy tumour recurrence due to differential uptake of tracer
Cherk et al. [155]	In 21 patients with NSCLC, low FMISO uptake was seen, with no correlation with FDG uptake and surrogate tissue markers of hypoxia, such as microvessel density and GLUT1 and angiogenesis
Gagel et al. [260]	In 8 patients with NSCLC treated with a combination of chemotherapy and/or radiotherapy, a decrease in FDG and FMISO uptake after treatment was associated with a favourable outcome, and a high initial FMISO uptake was a poor prognostic indicator
Vera et al. [156]	FMISO uptake higher in tumours than in nodes and did not change during therapy
Thureau et al. [245]	Low reproducibility and inter-observer agreement for FMISO volume measurements on the basis of visual assessment
Francis et al. [222]	Visual analysis demonstrated tumour FMISO activity in 17 of 20 patients with malignant mesothelioma. This pilot study confirmed that mesothelioma is a tumour with significant areas of hypoxia, particularly in dominant tumour masses
FAZA	
Bollineni et al. [164]	FAZA PET is able to detect heterogeneous distributions of hypoxic sub-volumes on visual analysis. No significant correlation between FAZA uptake and FDG SUVmax or lesion size
Trinkhaus et al. [165]	11/17 patients had baseline hypoxia, 6/8 patients with scans following chemoradiation had resolution of hypoxia based on qualitative assessment
HX4	
Zegers et al. [174]	In NSCLC patients, image contrast was found to be superior 4 h p.i. compared with earlier time points and uptake patterns were strongly correlated between two scans
FETNIM	
Li l et al. [177]	FETNIM tumour to blood ratio and hypoxic volume were strong predictors for overall survival. No correlation between FETNIM and FDG uptake
Hu m et al. [178]	FETNIM uptake was higher in tumours than in normal tissue. Similar data observed at 60 and 120 min p.i
FRP-170	
Kaneta et al. [182]	FRP-170 accumulation in normal lung shows stable tumour to blood ratio at 60–120 min p.i. Images may allow evaluation of tumour accumulation in a clinical setting
Cu-ATSM	
Dehdasthi et al. [261]	Imaging with [^60^Cu]Cu-ATSM is feasible in NSCLC. In 14 patients with biopsy-proven NSCLC, [^60^Cu]Cu-ATSM uptake predicted response to radiation or chemotherapy
Lohith et al. [232]	FDG and [^62^Cu]Cu-ATSM had spatially similar distributions in adenocarcinomas

Gastrointestinal tract cancers	
FMISO	
Roels et al. [239]	Mismatch between FDG and FMISO scans. FMISO uptake reduced after therapy
Segard et al. [241]	FMISO accumulation was observed in 2/10 patients with pancreatic cancer on the basis of visual analysis
FAZA	
Havelund et al. [170]	[^18^F]FAZA-PET is feasible for visualisation of hypoxia in rectal cancer
Nascente et al. [234]	In patients with pancreatic cancer FAZA PET imaging of hypoxia revealed a range of hypoxic fractions which correlated with pimonidazole staining. These preliminary results provide evidence of clinical feasibility and utility of FAZA PET in pancreatic cancer
FETNIM	
Yue et al. [180]	In this study, 11 of 14 tumours with FETNIM uptake responded poorly and only 1 of 14 tumours without uptake failed. High baseline SUVmax associated with poor clinical response
Cu-ATSM	
Dietz et al. [262]	Median tumour-to-muscle activity ratio of 2.6 discriminated those with worse prognosis from those with better prognosis in patients with rectal cancer. Overall and progression-free survival worse in hypoxic tumours

Genitourinary/gynaecological cancers	
FMISO	
Lawrentschuk et al. [228]	In a recent study correlating FMISO uptake with direct pO2 histographic measurements in 17 patients with renal cell carcinoma, the degree of FMISO uptake correlated with low tissue oxygen tension. Mild FMISO uptake may reflect renal tumour oxygenation of >10 mmHg
Hugonet et al. [158]	Reduction in hypoxic volume post-therapy
Rasey et al. [137]	The first clinical evidence to suggest hypoxia in prostate cancer came from a small study of four patients who underwent FMISO PET imaging
FAZA	
Schuetz et al. [169]	5/15 patients with cervical cancer had visually identifiable tumours
Garcia-Parra et al. [223]	FAZA uptake was not increased in prostate tumours as seen with CA9 immunohistochemistry staining
EF5	
Lin et al. [231]	In a pilot study of 8 patients with cervical carcinoma undergoing chemo-radiotherapy, TMR of >1.35 was shown to predict development of metastatic disease
FETNIM	
Vercellino et al. [179]	High uptake associated with lower progression-free and overall survival in patients with cervical cancer
Cu-ATSM	
Dehdasthi et al. [125]	Tumour uptake of [^60^Cu]Cu-ATSM inversely related to progression-free survival and overall survival. No correlation between FDG and [^60^Cu]Cu-ATSM uptake
Grigsby et al. [263]	In 15 patients with cancer of the uterine cervix who were imaged with [^60^Cu]-ATSM, hypoxia as determined by the PET images was a significant independent predictor of tumour recurrence. Fouryear overall survival estimates were 75% for patients with non-hypoxic tumours and 33% for those with hypoxic tumours
Lewis et al. [230]	Cu-ATSM uptake in 10 patients with cancer of the uterine cervix correlates with prognosis and patient outcome. The uptake pattern was reproducible with two scans 1–9 days apart, suggesting that the microscopic distribution of chronic hypoxia did not change greatly over this interval
Dehdasthi et al. [221]	Tumour uptake of [^60^Cu]-ATSM was inversely related to progression-free survival and cause-specific survival. 3-year progression-free survival of patients with non-hypoxic tumours was 71%, and 28% for those with hypoxic tumours

Soft tissue sarcoma	
FMISO	
Rajendran et al. [238]	In 19 soft-tissue sarcoma (STS) patients FMISO uptake has been correlated with VEGF expression, although there was no correlation between tumour grade, hypoxic volume, and FDG uptake
Bentzen et al. [220]	In a further study of 13 patients with soft-tissue tumours (7 confirmed malignant tumours and 6 benign tumours), no correlation between FMISO uptake and pO2 measurements was found
FAZA	
Lewin et al. [229]	Evaluated the implications of hypoxia in STS, using FAZA PET. Hypoxia was associated with radioresistance, higher local recurrence showing a poor outcome

### Identification of tumour hypoxia and prediction of prognosis/response to treatment

The clinical significance of hypoxia PET imaging is to identify individuals with poor prognosis and those likely to benefit from hypoxia-targeted therapy. Several studies have shown that hypoxia PET imaging predicts outcome. High FMISO retention has been associated with higher risk of loco-regional failure and shorter progression-free survival in head-and-neck [142, 248, 249, 252–254] and renal cancer [158]. Furthermore, a meta-review of the clinical data of over 300 patients concluded that FMISO is a predictor of poor treatment response and prognosis [131]. Similarly, FETNIM uptake in lung [176], head-and-neck [175], and oesophageal cancer [180], were also associated with poor outcomes. Studies conducted with FAZA in squamous cell carcinomas of the head and neck [167] and Cu-ATSM in patients with cervical [125, 261, 263], lung [125, 261], and rectal cancer [262] have shown that lower tumour-to-muscle ratio (TMR) is indicative of better prognosis.

These findings have been discussed in a recent meta-analysis of PET hypoxia studies which have demonstrated a common tendency towards predicting outcome in tumours showing higher tracer accumulation [162]. Decreased FMISO uptake with treatment has been widely reported in brain [152], head-and-neck [250, 255], lung [258, 260], and renal tumours [158]; although this was not seen in some tumours [142, 156]. Decrease in semi-quantitative imaging parameters such as tumour-to-muscle ratios (TMRs) signifying response to chemotherapy have also been demonstrated with Cu-ATSM in lung [125, 261] and head-and-neck tumours [257], and FAZA in lung cancer [165].

### Radiotherapy planning

It is well known that tumours demonstrate temporal changes and/or heterogeneity in the spatial distribution of hypoxia. Identification of these areas with PET hypoxia scans enables image guidance and hence, radiation dose escalation to radioresistant sub-volumes [162, 248, 259]. Boosting the dose to intra-tumoural areas of biological resistance (dose painting) is being pursued as a strategy to overcome radioresistance and improve outcomes [264]. This is made possible due to the advances in imaging and radiation treatment planning. The feasibility of this strategy has been investigated in cancers of the head and neck, lung, and brain with Cu-ATSM [256], FMISO [251], and FAZA [166], mostly on anthrophomorphic phantoms [166, 249, 251], and further studies are required for translation into clinical benefit.

## Discussion and concluding remarks

Hypoxia research has a long history; however, accurate and reproducible measurement of clinically relevant hypoxia with high sensitivity continues to evade the scientific community. Although radionuclide measurements of hypoxia started in the early 1980s we are yet to have a widely accepted method. In addition to studies with oxygen electrodes, imaging utilising exognous probes including FMISO-PET, FAZA-PET, HX4, and immunohistochemistry with pimonidazole have been the mainstay of hypoxia assessment in clinical studies; the EU-funded METOXIA consortium for example utilises HX4 for assessment of hypoxia [265]. In the post-genome era, genetic methods are also making an important entrance—with a 26-gene signature in validation for assessing hypoxia [266]. There have been some successes in the use of hypoxia measurements as part of clinical trials: a number of studies confirmed that hypoxia predicts locoregional failure to radiotherapy [81, 267, 268] and chemoradiotherapy with hypoxia-modulated cytotoxin tirapazamine [249]. Conversely, there have been several challenges, in particular poor sensitivity of the methods requiring long assessment periods (as pertains to nitroimidazole PET methods), invasive nature (as pertains to pimonidazole immunohistochemistry), an inability to relate measured output to oxygen tension (as pertains to MRI methods despite high spatial resolution) and wide heterogeneity within and between the tumours of the same patients and temporally with treatment (all methods).

Notably, despite design of newer nitroimidazoles with substantially different physicochemical properties—high hydrophilicity—concomitant improvements in signal-to-noise ratio and, thus, reduction in imaging times, have not been achieved, indicating that the ideal chemical design has not yet been realised. There is also paucity of studies examining heterogeneity of hypoxia using parametric imaging to detect the influence of hypoxia sub-volumes and whether this additional detail will have prognostic or predictive value. With the advent of PET-MRI scanners, it will be feasible to multiplex imaging modalities to provide addition information such as perfusion to increase accuracy of hypoxia measurements or provide complementary information with higher predictive value. Whatever selected method will require assessment of precision of measurement which is non-trivial with such a spatio-temporally evanescent phenomenon as hypoxia.

Thus, it is accepted that hypoxia could have significant prognostic and predictive value in the clinic; however, the best method for hypoxia assessment has in our opinion not been realised.
